# Sleep Apnea and Atrial Fibrillation: Clinical Features and Screening Diagnostic Options

**DOI:** 10.3390/jpm14060618

**Published:** 2024-06-09

**Authors:** Azamat Maratovich Baymukanov, Yuliya Dmitrievna Weissman, Irina Andreevna Bulavina, Ilya Leonidovich Ilyich, Sergey Arturovich Termosesov

**Affiliations:** Moscow City Clinical Hospital after V.M. Buyanov, Moscow 115516, Russia; judy50@mail.ru (Y.D.W.); doctoroirb@yandex.ru (I.A.B.); iilyich@mail.ru (I.L.I.); stermosesov@list.ru (S.A.T.)

**Keywords:** obstructive sleep apnea, atrial fibrillation, heart failure, screening, STOP-BANG scale, Berlin questionnaire

## Abstract

Introduction: Obstructive sleep apnea (OSA) is associated with an increased risk of hypertension, coronary artery disease, heart failure (HF), and atrial fibrillation (AF). Materials and methods: A total of 179 patients aged 34–81 years were included in the study. The median age was 63 years (interquartile range: 56–69 years). Of these patients, 105 (58.7%) were men, and 74 (41.3%) were women; there were cases of paroxysmal (*n* = 99), persistent (n = 64), and permanent AF (*n* = 16). All patients underwent investigations including respiratory sleep monitoring, echocardiography, and 24 h Holter electrocardiography monitoring. Statistical analyses were performed using IBM SPSS Statistics 26.0. Results: OSA was detected in 131 (73.2%) patients. In patients with OSA, paroxysmal AF was commonest (*n* = 65), followed by persistent AF (*n* = 51) and permanent AF (*n* = 15). The patients with sleep apnea had increased body mass index (33.6 kg/m2; *p* = 0.02), waist circumference (114 cm; *p* < 0.001), and neck circumference (42 cm; *p* < 0.001) values. HF (OR 2.9; 95% CI: 1.4–5.9; *p* = 0.004) and type 2 diabetes (OR 3.6; 95% CI: 1.5–8.3; *p* = 0.001) were more common in patients with AF and OSA. The STOP-BANG scale (AUC = 0.706 ± 0.044; 95% CI: 0.619–0.792; *p* < 0.001) and the Berlin questionnaire (AUC = 0.699 ± 0.044; 95% CI: 0.614–0.785) had a higher predictive ability for identifying sleep apnea. Conclusions: Patients with AF demonstrate a high prevalence of OSA and an increased association with cardiovascular comorbidities. The STOP-BANG scale and the Berlin questionnaire can be used to screen for OSA in patients with AF.

## 1. Introduction

Obstructive sleep apnea (OSA) is a common disorder that can manifest with or without symptoms and is associated with serious neurocognitive and cardiovascular consequences. Recent data indicate that sleep apnea may affect 730 million people worldwide [1]. Patients with OSA experience recurrent breathing pauses during sleep, leading to periodic nighttime hypoxemia, hypercapnia, increased sympathetic nervous system activation, elevated oxidative stress, and an enhanced inflammatory response. These factors contribute to atrial remodeling and fibrosis, thereby predisposing patients to an increased risk of developing and/or maintaining atrial fibrillation (AF) [2].

AF is a well-recognized contributor to various adverse outcomes, including ischemic stroke and heart failure (HF). OSA and AF share common risk factors and both result in a significant reduction in quality of life. Some studies suggest that sleep apnea is detected in approximately 50% of patients with AF [3]. OSA also reduces the likelihood of maintaining sinus rhythm after electrical cardioversion or catheter ablation. AF imposes a substantial economic burden on several countries. AF-associated healthcare expenditure is projected to reach USD 45.4 billion in the United States by 2030 [4]. Given the significance of this issue, there is a need for diagnostic screening for OSA. The most straightforward and accessible method for the non-instrumental detection of sleep apnea is through questionnaire completion. Three questionnaires have gained widespread use: STOP-BANG, the Berlin questionnaire, and the Epworth Sleepiness Scale. Considering the interrelationship between AF and OSA, the presence of shared risk factors, and their high impact on the course of comorbid conditions, we conducted a study aimed at investigating the prevalence of OSA, as well as anthropometric and clinical–instrumental characteristics of patients with AF suffering from sleep-disordered breathing.

## 2. Materials and Methods

This study was a prospective observational one. Patients with atrial fibrillation were selected for catheter isolation of the pulmonary veins by cardiologists during outpatient visits. Patient recruitment took place over a period of 12 months.

The study included 179 patients aged 34 to 81 years old. The median age was 63 years (interquartile range: 56 to 69 years). A total of 105 (58.7%) patients were men, and 74 (41.3%) were women; the patients were suffering from either the paroxysmal (*n* = 99; 55.3%), persistent (*n* = 64; 35.8%), or permanent forms of AF (*n* = 16; 8.9%).

The inclusion criteria were as follows:

- Patients aged 18 to 80 years old.
- Confirmed diagnosis of AF.
- Signed informed consent form to participate in the study.

The exclusion criteria were as follows:

- Myocardial infarction within the past 3 months prior to study inclusion.
- Concomitant severe somatic diseases (endocrine pathology, renal and/or hepatic insufficiency, oncological diseases) with an estimated life expectancy of less than 1 year.
- Pregnancy or breastfeeding.
- Mental illness (severe dementia, schizophrenia, severe depression, manic-depressive psychosis).

All patients underwent laboratory and instrumental investigations, including respiratory sleep monitoring (SOMNOtouch RESP eco, SOMNOmedics GmbH, Randersacker, Germany), echocardiography (GE E90, USA), and 24 h Holter ECG monitoring.

Respiratory monitoring was performed during overnight sleep lasting at least 7 h. Each patient’s nasal airflow through nasal cannulas, oxygen saturation, chest excursion, body position, snoring episodes, and heart rate were recorded. Patients’ body position was determined using an integrated body position sensor (five positions: right side, left side, supine, prone, vertical). Activity was detected using an integrated motion sensor which recognized motion artifacts along three axes. The polysomnograph was secured to a patient’s body using flexible straps, and leads from the pulse oximeter and nasal cannulas were secured with adhesive tape.

The study utilized automatic device activation/deactivation to minimize the risk of recording failure. The main parameters for diagnosing sleep apnea and determining its severity included the following: sleep efficiency, number of apnea and hypopnea episodes, apnea–hypopnea index (AHI), desaturation index (average number of apnea episodes per hour of sleep with oxygen saturation decreasing by more than 4% from baseline), maximum and average apnea duration, number and duration of episodes with oxygen saturation less than 90% and 80%, and snoring episodes.

Preliminary screening for the presence of sleep apnea was conducted using the STOP-BANG, Berlin, and Epworth Sleepiness Scale questionnaires. The STOP-BANG questionnaire consisted of two parts, STOP and BANG, and also assessed the risk of OSA based on the presence of snoring, daytime sleepiness, observed apnea by others, elevated blood pressure, body mass index (BMI), age, neck circumference, and gender. A high risk of OSA was determined when the number of “Yes” answers was 5–8. Additional conditions for assessing possible high risk included “Yes” to 2 or more of the 4 STOP questions + male gender or “Yes” to 2 or more of the 4 STOP questions + BMI > 35 kg/m^2^ or “Yes” to 2 or more of the 4 STOP questions + neck circumference ≥ 40 cm. Medium risk of OSA was defined as 3–4 “Yes” answers, and low risk was defined as 0–2 “Yes” answers.

The Berlin questionnaire assessed the presence of snoring, fatigue, and tiredness after sleep, as well as the presence of hypertension and BMI value. The criterion for the severity of sleep apnea when assessing the results of the Berlin questionnaire was a positive value in two or more categories, indicating a high likelihood of sleep apnea in this patient. The Epworth Sleepiness Scale assessed the presence and severity of daytime sleepiness. It described various day-to-day situations in which a person had the possibility of falling asleep. Increased daytime sleepiness; low sleep quality; irritability; inattention; and the likelihood of falling asleep while reading a book, watching TV, or talking could indicate the presence of sleep apnea.

### Statistical Analysis

Statistical analysis was performed using IBM SPSS Statistics software, version 26. Normality of distribution was assessed using the Kolmogorov–Smirnov test. Since the investigated quantitative variables did not exhibit a normal distribution, non-parametric tests were used for statistical calculations. Quantitative variables are presented as median and interquartile range. The Mann–Whitney U test was applied to assess differences in continuous variables between two independent samples. Contingency tables with Pearson’s chi-square (χ^2^) criterion assessment, odds ratio (OR), and confidence interval (CI) calculations were used to test hypotheses about the independence of categorical features. The Spearman rank correlation coefficient was applied to detect and assess the strength of the relationship between two sets of comparable quantitative variables. The Kruskal–Wallis test was used to test the equality of medians of multiple samples. The receiver operating characteristic (ROC) curve method was used for binary classification results and to determine the quality of the prognostic model. A *p*-value < 0.05 was considered statistically significant.

## 3. Results

According to the respiratory monitoring conducted, OSA was detected in 131 (73.2%) patients. Among them, mild OSA was found in 39 (29.8%) patients, moderate OSA in 49 (37.4%), and severe OSA in 43 (32.8%). In the observed group of patients with identified OSA, the paroxysmal form of AF predominated (*n* = 65, 49.6%), followed by persistent AF (*n* = 51, 38.9%) and permanent AF (*n* = 15, 11.5%). The main anthropometric indicators of the overall patient sample (*n* = 179) were calculated. The median BMI value was 33.4 kg/m^2^, that for waist circumference was 112 cm, and that for neck circumference was 42 cm. Additionally, Table 1 presents the main indicators of patients with and without OSA.

When comparing the anthropometric data of patients with and without OSA, statistically significant differences were found in age, weight, BMI, waist circumference, and neck circumference. Among the investigative parameters, differences were observed only in the level of HDL cholesterol.

**Figure 1:**
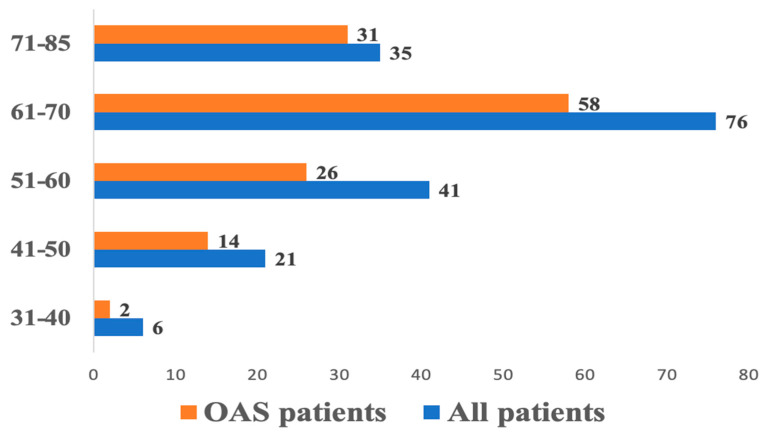
Distribution of patients with OSA by 10-year age groups.

After categorizing patients into 10-year age groups (except the group of patients older than 70 years), ranging from 30 to 85 years, a gradual increase in the proportion of patients with OSA was observed (Figure 1).

The anthropometric data of patients, stratified by the severity of sleep apnea, are presented in Table 2. When evaluating laboratory and ultrasound parameters, statistically significant differences were found only in the glomerular filtration rate (also presented in Table 2).

There were statistically significant differences in body weight among patients depending on the severity of OSA (*p* = 0.021). When comparing the groups pairwise, it was found that body weight was significantly higher in those with moderate and severe degrees of sleep apnea compared to those with mild sleep apnea (*p* = 0.047 and *p* = 0.041, respectively). A statistically significant difference in BMI was also observed (*p* = 0.014): BMI was significantly lower in those with mild OSA compared to those with severe OSA (*p* = 0.010). Waist circumference also differed among the subgroups: it was significantly smaller in those with mild OSA compared to those with the moderate or severe forms of OSA (*p* = 0.04 and *p* = 0.001, respectively).

The presence of hypertension, HF, history of myocardial infarction, and diabetes was compared between patients with and without OSA. The results are presented in Table 3.

When comparing the frequency of HF depending on the presence of OSA, statistically significant differences were obtained (*p* = 0.04). Patients with OSA were 2.9 times more likely to develop HF (95% CI: 1.4–5.9; *p* = 0.004). A moderate association was observed between the compared variables (V = 0.22).

Patients with OSA were 3.6 times more likely to develop type 2 diabetes (95% CI: 1.5–8.3; *p* = 0.001). Similarly, a moderate association was observed between the compared variables (V = 0.23).

The severity of OSA is determined by the AHI. At the same time, the results of respiratory sleep monitoring consist of various parameters that provide a practically complete understanding of the patient’s sleep. An evaluation of sleep characteristics was performed on patients with OSA of different severities. The sleep efficiency of patients with different severities of apnea was high. Significant differences in most sleep parameters, such as AHI, maximum apnea duration, desaturation index, minimum saturation, and the number of snoring episodes were expected. This was especially evident between patients with mild and severe OSA. The central apnea index was not significantly different, suggesting a greater contribution of obstructive mechanisms to the occurrence of sleep apnea in patients with HF.

All patients completed the STOP-BANG, Berlin, and Epworth questionnaires the day before sleep apnea diagnosis. According to the STOP-BANG questionnaire, OSA was suspected with a high probability in 125 (69.8%) patients; according to the Berlin questionnaire, it was suspected in 124 (69.3%) patients, and according to the Epworth scale, it was suspected in 24 (13.4%) patients.

Statistically significant differences in the frequency of OSA among patients were obtained depending on the results of the STOP-BANG and Berlin questionnaires. The likelihood of having sleep apnea for patients with five or more points (plus additional criteria) based on using the STOP-BANG scale increased by 3.3 times, and according to the Berlin questionnaire, it increased by 5.3 times (95% CI: 1.67–6.76; and 95% CI: 2.6–11.02; both *p* < 0.001, respectively). A moderate association was observed between the compared variables based on the STOP-BANG scale (V = 0.26). A moderate association was also observed between the compared variables based on the Berlin questionnaire (V = 0.36). Detailed data are presented in Table 4

**Figure 2:**
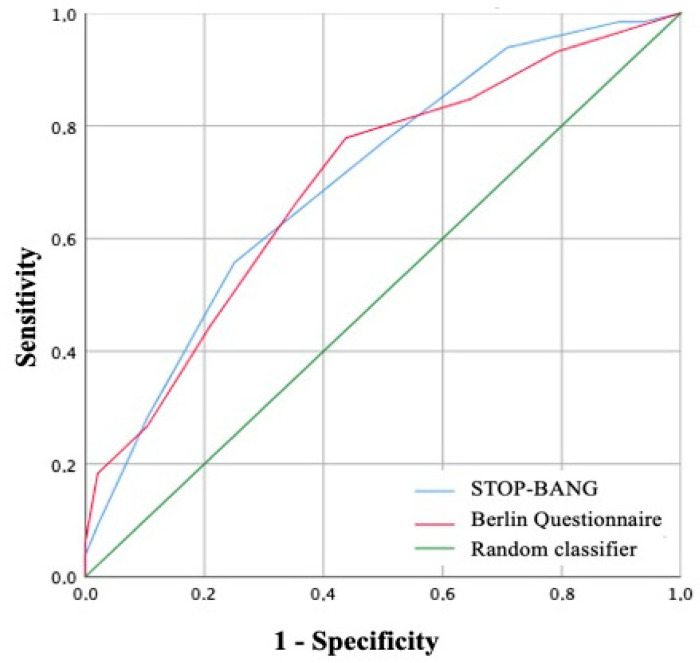
ROC curve corresponding to the relationship between predictive results of the Berlin questionnaire, the STOP-BANG scale, and OSA.

The diagnostic significance of the STOP-BANG and Berlin questionnaires in predicting OSA was evaluated using ROC curve analysis (Figure 2). The area under the ROC curve corresponding to the relationship between the STOP-BANG questionnaire results and OSA was 0.706 ± 0.044, with a 95% confidence interval of 0.619–0.792; *p* < 0.001. The sensitivity and specificity of the method were 77.1% and 50%, respectively. The area under the ROC curve corresponding to the relationship between the Berlin questionnaire results and OSA was 0.699 ± 0.044, with a confidence interval of 0.614–0.785; *p* < 0.001. The sensitivity and specificity of the method were 66.4% and 65%, respectively.

## 4. Discussion

The results of our study on the anthropometric and clinical data of patients with AF and OSA have been presented. This study demonstrated a significant prevalence of OSA among these patients. The findings exceed the frequency of OSA in other populations but are consistent with previously reported results in studies of patients with AF. Predominance of moderate and severe OSA (49 (37.4%) and 43 (32.8%), respectively) was identified. Considering that the diagnosis of sleep apnea was made for the first time in these patients, this may suggest that the issue is underestimated by cardiologists and that there is a low level of awareness about it in society.

Of note is the increase in the occurrence of OSA with advancing age, which is comparable to the increased likelihood of detecting AF at each subsequent decade of life [5]. Patients with OSA were expectedly found to have higher body mass and, consequently, a higher BMI. Obesity significantly contributes to the progression of left atrial remodeling [6,7]. Data from a cohort study (*n* = 3542) demonstrated that in the subgroup of patients <65 years old, obesity, age and nocturnal desaturation, were predictors of newly diagnosed AF [8]. Additionally, our data revealed statistically significant differences in waist and neck circumference.

When grouped by sleep apnea severity, almost equal numbers of patients with mild, moderate, and severe disease were identified. However, the number of patients with an AHI greater than 15 per hour (indicative of moderate to severe OSA) amounted to 84 (76.3% of patients with identified OSA and 46.9% of the total number of patients). Such a large number of patients with moderate to severe OSA validates the hypothesis provided by Mehra R. et al. regarding the increased risk of AF with increasing severity of sleep apnea [9]. In our study, OSA predominated over central sleep apnea, which is also expected for patients with AF, and this has been confirmed in other studies [2]. Significant differences in estimated glomerular filtration rate (eGFR) were observed in the laboratory data. The median eGFR values in patients with mild and severe OSA were 72 (59.5–85.4) and 61.6 (54–72) mL/min/1.73 m^2^, respectively (*p* = 0.025). This decrease in eGFR in patients with severe OSA may be explained by the potential impact of apnea on kidney function. Epidemiological studies indicate a causal relationship between OSA and CKD, while experimental data further confirm that OSA induces CKD due to elevated blood pressure, oxidative stress, and hypoxia [10].

According to various studies, OSA is considered a risk factor for hypertension, HF, transient ischemic attack/stroke, and diabetes [11,12,13,14]. The important relationship between hypertension and OSA has been noted in recent European guidelines. In a published document, OSA was mentioned approximately 50 times and received special attention in a separate paragraph under the heading “Hypertension and other individual comorbidities” [11]. In our study, patients with hypertension prevailed in each group, but no significant difference was found between them (*p* = 0.087). According to Polecka A. et al., OSA is closely associated with adverse outcomes in patients with HF [15]. Patients with sleep apnea also presented more frequently with HF and type 2 diabetes (OR 2.9; 95% CI: 1.4–5.9 (*p* = 0.004) and OR 3.6; 95% CI: 1.5–8.3; *p* = 0.001, respectively). Holt A. et al. corroborate our data that patients over 60 years old diagnosed with OSA have a significantly increased risk of developing HF [16]. Furthermore, a systematic review of 41 studies in adults with type 2 diabetes showed a prevalence of OSA in 60% of cases [17]. A meta-analysis of 16 cohort studies with 338,912 participants showed that among 19,355 individuals who developed type 2 diabetes over a mean follow-up period of 10.5 years, the relative risk was 1.4 times higher in those with OSA [18]. The high prevalence of HF among patients with OSA and AF contributes to the development of comorbidities and may worsen the course and prognosis of this group.

In our study, we evaluated the application of three questionnaires to detect OSA in patients with AF: the STOP-BANG questionnaire, the Berlin questionnaire, and the Epworth Sleepiness Scale (ESS). The STOP-BANG and Berlin questionnaires demonstrated high screening effectiveness. According to the ROC analysis, the Berlin questionnaire showed greater sensitivity and specificity compared to the STOP-BANG questionnaire. According to a systematic review by Amra B. et al., the STOP-BANG questionnaire has the highest sensitivity for predicting mild and severe OSA (97.55% and 98.7%, respectively), while the Berlin questionnaire showed the highest specificity for detecting mild and severe OSA (90% and 80%, respectively) [19]. The low effectiveness of the ESS as a tool for preliminary OSA diagnosis may be due to the specificity of the patient group with AF. In a similar study conducted by Traaen G. et al., only 20.9% and 17.4% of patients diagnosed with moderate and severe OSA, respectively, had 11 or more points on the Epworth Sleepiness Scale [20]. The Epworth Sleepiness Scale evaluates daytime sleepiness, which is an important symptom of OSA, but it is present in only 28–35% of patients with an AHI ≥ 15 events/hour [5]. In the population of patients with AF who underwent electrical cardioversion, 24% reported excessive daytime sleepiness, while the sensitivity and specificity of the Epworth Sleepiness Scale at the AHI threshold of 15 events/hour were 29.1% and 58.3%, respectively [21]. In other studies, the scale also showed low effectiveness in patients with various forms of AF (AUC < 0.6) [20,22]. Considering the results of our study, it can be concluded that self-reported daytime sleepiness poorly correlates with AHI and that the Epworth Sleepiness Scale is not an acceptable tool for detecting OSA in patients with AF. At the same time, the STOP-BANG and Berlin questionnaires demonstrate high sensitivity and insufficient specificity, requiring further modification.

## 5. Conclusions

This study presented the anthropometric and clinical characteristics of patients suffering from AF and OSA. A high frequency of OSA was observed in this patient group. Patients with sleep apnea exhibited more pronounced obesity and greater neck and waist circumferences. Among patients with OSA and AF, HF and diabetes were more frequently diagnosed, directly impacting the formation of multimorbidity in these patients. The Epworth Sleepiness Scale is not an effective screening method for diagnosing OSA in patients in this group. The Berlin and STOP-BANG questionnaires can be used to assess the risk of OSA in patients with AF with high sensitivity. However, these screening tools require improvements to enhance their specificity.

## 6. Study Limitations

(1) This study was not population-based.

Included patients with atrial fibrillation (AF) who underwent respiratory sleep monitoring were initially referred to the interventional cardiology department for pulmonary vein isolation, electrical cardioversion, or pacemaker implantation. Therefore, this patient group may not be representative of the general population of AF patients receiving pharmacological treatment on an outpatient basis.

(2) Patients were initially classified as being at high risk of developing OSA. The median body mass index (BMI) value of the patients included in the study was 33.4 kg/m^2^, which already classified them into obesity grade 1. Since obesity is a significant risk factor for OSA, our study may have resulted in inflated values for the prevalence of OSA and the anthropometric characteristics of patients with AF. This limitation could potentially be mitigated by comparisons with the BMI values of patients without AF.

## Figures and Tables

**Table 1:** Characteristics of patients with and without OSA.

Characteristics	With OSA (*n* = 131)	Without OSA (*n* = 48)	*p* Value
Male, *n* (%) Female, *n* (%)	82 (62.6%) 49 (37.4%)	23 (47.9%) 25 (52.1%)	0.079
Age (years)	65 (58–70)	57 (51.2–66)	0.001
Height (cm)	173 (165.5–178.5)	174 (164–178.5)	0.812
Weight (kg)	98 (88.5–112.6)	92.5 (82.5–105)	0.025
BMI (kg/m^2^)	33.6 (30.3–37.9)	31.3 (27.5–35.2)	0.020
Abdominal circumference (cm)	114 (108–124.5)	107.5 (100–112)	<0.001
Neck circumference (cm)	42 (40.8–46)	41 (38–42.5)	<0.001
Hemoglobin (g/L)	146 (137–159.5)	148 (138–160)	0.839
Glycated hemoglobin (%)	6.1 (5.7–6.6)	5.9 (5.4–6.4)	0.118
GFR (mL/min/1.73 m^2^)	64 (55.15–76.8)	71.15 (61.4–78.5)	0.367
HDL cholesterol (mmol/L)	1.08 (0.89–1.22)	1.15 (0.97–1.36)	0.011
LDL cholesterol (mmol/L)	2.11 (1.53–2.71)	2.37 (1.82–3.13)	0.831
CHA2DS2-VASc (score)	2.5 (2–3)	2 (1–3)	0.085
LV EDV	110 (95–128)	110 (98–129)	0.892
LV ejection fraction (%)	55 (48.35–61.75)	56 (55–63)	0.154
LA AP	42.9 (40–46.25)	42 (39.7–45)	0.229
LAVI	34.99 (18.3–43.1)	37.35 (21.6–45.7)	0.667

GFR—glomerular filtration rate, HDLs—high-density lipoproteins, LDLs—low-density lipoproteins, EDV—end diastolic volume, LV—left ventricle, LA AP—left atrium anterior–posterior dimension, LAVI—left atrial volume indexed.

**Table 2:** Comparative characteristics of patients with different degrees of OSA.

Characteristics	Mild OSA (*n* = 39)	Moderate OSA (*n* = 49)	Severe OSA (*n* = 43)	*p* Value
Age (Years)	64 (57–70)	66 (60–71)	66 (55–70)	0.342
Height (cm)	172 (163–178)	175 (168–180)	174 (165.5–178)	0.347
Weight (kg)	93 (86–103)	102 (95–115)	105 (87.5–120.5)	0.021 *p*_1–2_ = 0.047 *p*_1–3_ = 0.041 *p*_2–3_ = 1.000
BMI (kg/m^2^)	31.9 (28.1–35.1)	34.1 (30.9–38)	35.5 (31.9–39.7)	0.014 *p*_1–2_ = 0.374 *p*_1–3_ = 0.010 *p*_2–3_ = 0.385
Abdominal circumference (cm)	110 (101–118)	114 (109–127)	118 (110–128.5)	0.001 *p*_1–2_ = 0.040 *p*_1–3_ = 0.001 *p*_2–3_ = 0.492
Neck circumference (cm)	42 (40–45)	42 (41–45)	44 (40.8–48.5)	0.062
GFR (mL/min/1.73 m^2^)	72 (59.5–85.4)	65.2 (57–75.2)	61.6 (54–72)	0.027 *p*_1–2_ = 0.179 *p*_1–3_ = 0.025 *p*_2–3_ = 1.000

**Table 3:** Comparison of patients with and without OSA by comorbidities.

Disease	With OSA		Without OSA		*p* Value	OR; 95% CI
	Absolute	%	Absolute	%		
Hypertension	117	75.5	38	24.5	0.087	2.9; 0.9–5.3
Heart failure	68	84	13	16	0.004	2.9; 1.4–5.9
Myocardial infarction	10	90.9	1	9.1	0.293	3.8; 0.48–31.1
Type 2 diabetes	55	87.3	8	12.7	0.001	3.6; 1.5–8.3

**Table 4:** Relationship between questionnaire results and the presence of OSA.

Questionnaires/Scales	With OSA		Without OSA		*p* Value	OR; 95% CI
	Absolute	%	Absolute	%		
STOP-BANG	101	77.1	24	50	<0.001	3.3; 1.67–6.76
Berlin	104	79.4	20	41.7	<0.001	5.3; 2.6–11.02
Epworth	19	14.7	5	10	0.473	1.5; 0.54–4.41

## Data Availability

The original contributions of this study are included in the article; further inquiries can be directed to the corresponding author.

## References

[1] Benjafield A.V., Ayas N.T., Eastwood P.R., Heinzer R., Ip M.S.M., Morrell M.J., Nunez C.M., Patel S.R., Penzel T., Pépin J.-L. (2019). Estimation of the global prevalence and burden of obstructive sleep apnoea: A literature-based analysis. Lancet Respir. Med..

[2] Linz D., McEvoy R.D., Cowie M.R., Somers V.K., Nattel S., Levy P., Kalman J.M., Sanders P. (2018). Associations of Obstructive Sleep Apnea with Atrial Fibrillation and Continuous Positive Airway Pressure Treatment A Review. JAMA Cardiol..

[3] Zhang L., Hou Y., Po S.S. (2015). Obstructive Sleep Apnoea and Atrial Fibrillation. Arrhythmia Electrophysiol. Rev..

[4] AHAS Update (2017). Heart Disease and Stroke Statistics—2021 Update: A Report from the American Heart Association. Circulation.

[5] Gottlieb D.J., Whitney C.W., Bonekat W.H., Iber C., James G.D., Lebowitz M., Nieto F.J., Rosenberg C.E. (1999). Relation of Sleepiness to Respiratory Disturbance Index the Sleep Heart Health Study. Am. J. Respir. Crit. Care Med..

[6] Tarzimanova A.I. (2022). Obesity and atrial fibrillation: Mechanisms of occurence and current treatment guidelines. Therapy.

[7] Zhao H., Huang R., Jiang M., Wang W., Chai Y., Liu Q., Tao Z., Wu Q., Yue J., Ma J. (2023). Myocardial Tissue-Level Characteristics of Adults with Metabolically Healthy Obesity. Cardiovasc. Imaging.

[8] Gami A.S., Hodge D.O., Herges R.M., Olson E.J., Nykodym J., Kara T., Somers V.K. (2007). Obstructive Sleep Apnea, Obesity, and the Risk of Incident Atrial Fibrillation. J. Am. Coll. Cardiol..

[9] Mehra R., Stone K.L., Varosy P.D., Hoffman A.R., Marcus G.M., Blackwell T., Ibrahim O.A., Salem R., Redline S. (2009). Nocturnal arrhythmias across a spectrum of obstructive and central sleep-disordered breathing in older men: Outcomes of sleep disorders in older men (MrOS sleep) study. Arch. Intern. Med..

[10] Abuyassin B., Sharma K., Ayas N.T., Laher I. (2015). Obstructive Sleep Apnea and Kidney Disease: A Potential Bidirectional Relationship? Journal of Clinical Sleep Medicine. Am. Acad. Sleep Med..

[11] Mancia G., Kreutz R., Brunström M., Burnier M., Grassi G., Januszewicz A., Muiesan M.L., Tsioufis K., Agabiti-Rosei E., Algharably E.A.E. (2023). 2023 ESH Guidelines for the Management of Arterial Hypertension The Task Force for the Management of Arterial Hypertension of the European Society of Hypertension: Endorsed by the International Society of Hypertension (ISH) and the European Renal Association (ERA). J. Hypertens..

[12] Javaheri S., Peker Y., Yaggi H.K., Bassetti C.L. (2022). Obstructive sleep apnea and stroke: The mechanisms, the randomized trials, and the road ahead. Sleep Med. Rev..

[13] Mc Farlane S.I. (2018). Obesity, obstructive sleep apnea and type 2 diabetes mellitus: Epidemiology and pathophysiologic insights. Sleep Med. Disord. Int. J..

[14] Javaheri S., Brown L.K., Abraham W.T., Khayat R. (2020). Apneas of Heart Failure and Phenotype-Guided Treatments: Part One: OSA. Chest.

[15] Polecka A., Olszewska N., Danielski Ł., Olszewska E. (2023). Association between Obstructive Sleep Apnea and Heart Failure in Adults—A Systematic Review. J. Clin. Med..

[16] Holt A., Bjerre J., Zareini B., Koch H., Tønnesen P., Gislason G.H., Nielsen O.W., Schou M., Lamberts M. (2018). Sleep apnea, the risk of developing heart failure, and potential benefits of continuous positive airway pressure (CPAP) therapy. J. Am. Heart Assoc..

[17] Khalil M., Power N., Graham E., Deschênes S.S., Schmitz N. (2020). The association between sleep and diabetes outcomes—A systematic review. Diabetes Res. Clin. Pract..

[18] Qie R., Zhang D., Liu L., Ren Y., Zhao Y., Liu D., Liu F., Chen X., Cheng C., Guo C. (2020). Obstructive sleep apnea and risk of type 2 diabetes mellitus: A systematic review and dose-response meta-analysis of cohort studies. J. Diabetes.

[19] Amra B., Rahmati B., Soltaninejad F., Feizi A. (2018). Screening questionnaires for obstructive sleep apnea: An updated systematic review. Oman Med. J..

[20] Traaen G.M., Øverland B., Aakerøy L., Hunt T.E., Bendz C., Sande L., Aakhus S., Zaré H., Steinshamn S., Anfinsen O.G. (2020). Prevalence, risk factors, and type of sleep apnea in patients with paroxysmal atrial fibrillation. IJC Heart Vasc..

[21] Albuquerque F.N., Calvin A.D., Kuniyoshi F.H.S., Konecny T., Lopez-Jimenez F., Pressman G.S., Kara T., Friedman P., Ammash N., Somers V.K. (2012). Sleep-disordered breathing and excessive daytime sleepiness in patients with atrial fibrillation. Chest.

[22] Kadhim K., Middeldorp M.E., Elliott A.D., Jones D., Hendriks J.M., Gallagher C., Arzt M., Jones D., Hendriks J.M.L., Gallagher C. (2019). Self-Reported Daytime Sleepiness and Sleep-Disordered Breathing in Patients with Atrial Fibrillation: SNOozE-AF. Can. J. Cardiol..

